# The Action of Quinine upon the Ear

**Published:** 1883-08

**Authors:** 


### The Action of Quinine Upon the Ear.

Green (*Boston Med. and Surg. Jour.*, March 8, 1883) has an interesting and timely paper upon the above subject, and formulates his conclusions as follows: 1. Clinical experience the world over is that quinine occasionally produces serious injury to the ears. 2. From our present knowledge, both clinical and experimental, we are justified in asserting that the action of quinine upon the ears is to produce congestion of the labyrinth and tympanum, and sometimes distinct inflammation, with permanent tissue changes. 3. The action of the drug upon the ears should always be considered in prescribing it, and changes in the ears, due to existing or previous inflammation of those organs, constitute a contraindication to the medicine in large doses or for a long time, except under urgent circumstances. 4. Where large and continuous doses are absolutely necessary, an occasional intermission of the administration is desirable, if possible, to diminish the risk to the ears.—*The New York Medical Journal.*

